# The complete mitochondrial genome of *Ixodes vespertilionis* (Acari: Ixodidae)

**DOI:** 10.1080/23802359.2021.1976686

**Published:** 2021-09-20

**Authors:** Xinyan Lu, Xiuhua Zuo, Dandan Jiang, Xing Yang

**Affiliations:** a Integrated Laboratory of Pathogenic Biology, College of Preclinical Medicine, Dali University, Dali, PR China; b Affiliated Hospital of Dali University, Dali Yunnan, PR China

**Keywords:** *Ixodes vespertilionis*, mitogenome, phylogeny

## Abstract

*Ixodes vespertilionis* is a tick parasitizing on the bodies of bats. In our study, the complete mitogenome of *I. vespertilionis* was determined by using Illumina sequencing technology. The mitogenome was 14,559 bp in size and was predicted to encode 37 genes including 13 protein-coding genes, 22 transfer RNA genes, 2 ribosomal RNA genes, and one control region. The gene order of the mitogenome is identical to Argasidae and non-Australasian Prostriata. The phylogenetic analysis by the Maximum-likelihood method reveals that *I. vespertilionis* is phylogenetically closest to *Ixodes simplex*. These data provide novel reference for further studies on the population genetics and phylogenetics of ticks.

*Ixodes vespertilionis* (Koch, 1844) (Acari: Ixodidae) mainly inhabits in caves and exclusively parasitizes bats, such as *Rhinolophus sinicus, Rhinolophus macrotis* and *Hipposideros pratti* (Bush and Robbins 2012). It has been recorded in various parts of the world including Asia, European and African countries (Hornok et al. 2015). In China, *I. vespertilionis* is mainly distributed in the Inner Mongolia, Jiangsu, Fujian, Sichuan, Hubei, Shanxi, Liaoning, Guizhou and Yunnan, and it also appears in Taiwan (Bush and Robbins 2012). Fragments of the mitochondrial genes encoding *COX1*, *COX2*, *16S* rRNA and *12S* rRNA of *I. vespertilionis* have already been sequenced and utilized for studies. However, the phylogenetic studies of this species are not clear enough due to the absence of sufficient genetic information.

In this study, we reported the complete mitogenome of *I. vespertilionis* for the first time. The *I. vespertilionis* were collected from bat cave which is located in Chuxiong City, Yunnan Province, China (25°01′N, 101°54′E) in August 2020. The ticks were collected from *Rhinolophus sinicus*. After collection (n = 2), one of them was kept as a voucher specimen and the other one was used for DNA extraction. The collected specimen was transported and stored at the Parasitological Museum, Dali University (Voucher number: DLUP2008). Species identification was conducted by Professor Chunhong Du that was based on morphological characterization. The sample was preserved in 95% ethanol and stored at −20 °C until utilization (Du et al. 2018). The genomic DNA was isolated by the standard phenol-chloroform extraction procedure (Burger et al. 2014). The whole-genome shotgun method was used in whole genome paired-end sequencing (Coil et al. 2015) and was conducted on the Illumina NovaSeq platform at Shanghai Personal Biotechnology Co, Ltd, Shanghai, China. The mitogenome of *I. vespertilionis* was assembled by using A5-miseq v20150522 software and SPAdesv3.9.0 software (Baker and Edlund 2020). Eventually, genome components annotation was retrieved by using the MITOS web server (Bernt et al. 2013) (http://mitos.bioinf.uni-leipzig.de/).

The mitogenome of *I. vespertilionis* was 14,559 bp in length and was submitted in GenBank under accession number: MW411447. The mitochondrial genome contained 13 protein-coding genes (PCGs), 22 transfer RNA genes (tRNAs), 2 ribosomal RNA genes (rRNAs) and one control region. The gene arrangement was identical to soft ticks and non-Australasian *Ixodes* species (Chen et al. 2020). Four of these PCGs (*NAD4*, *NAD4L*, *NAD1* and *NAD5*) were located on the light strand (L-strand), all other remaining PCGs were anchored on the heavy strand (H-strand) (Wang et al. 2020). We observed that the size of 22 transfer RNA genes ranging from 56 bp (tRNA-*Ser*) to 68 bp (tRNA-*Lys*), nine tRNA genes (tRNA-*Gln*, tRNA-*Cys*, tRNA-*Tyr*, tRNA-*Phe*, tRNA-*His*, tRNA-*Pro*, tRNA-*Leu1*, tRNA-*Leu2*, tRNA-*Val*) were encoded on the L-strand.

Among thirteen PCGs, *COX1*, *ND5, ND6* and *ND3* were begun with ATT, *ND2, ATP6* and *ND4* with ATA, only *ATP8* with ATC, the remaining five PCGs were characterized by ATG as the initiation codon. And most genes contained TAA as the termination codon, and *COX2*, *ND5, COX3* and *ND1* used the incomplete stop codon T (Guo et al. 2016). The general based composition of the mitogenome was A (38.15%), T (36.79%), C (16.47%), G (8.59%), with A + T (74.94%) having a higher ratio than G + C (25.06%). Meanwhile, no sequence identical to the Tick-Box consensus motif is present in the whole mtDNA of *I. vespertilionis* (Montagna et al. 2012).

Besides *I. vespertilionis,* we chose *Limulus polyphemus* (NC003057) as the outgroup and 18 other close species of the family *Ixodidae* to construct the phylogenetic tree. The evolutionary history was inferred by using the maximun likehood method based on the Tamura-Nei model with 1000 bootstrap replications. The nucleotide sequences of 13 protein-coding genes were analyzed in the final dataset. Evolutionary analyses were conducted in MEGA7.0 software (Kumar et al. 2016).

As shown in Figure 1, the result showed that this phylogenetic tree was divided into two large branches: Metastriata and Prostriata. Within the prostriata, *I. vespertilionis* was clustered together with species in the genus *Ixodes* and shared close relationship with *I. simplex,* supporting *I. vespertilionis* among the family Ixodidae (Charrier et al. 2019). In conclusion, the complete mitogenome of *I. vespertilionis* provides a new resource for phylogenetic studies and can be used as novel reference for further studies on *Ixodes* ticks.

**Figure 1.**
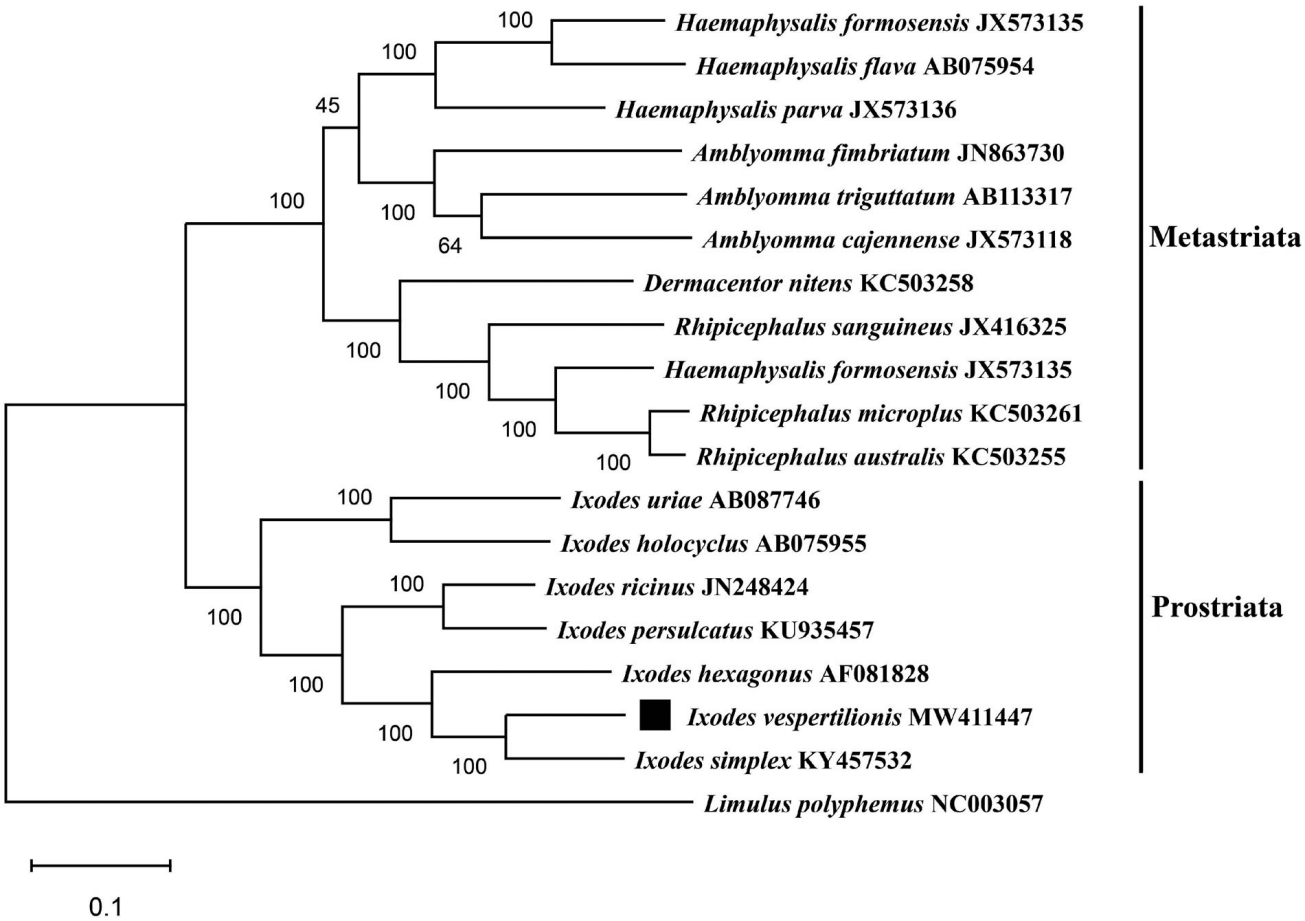
Maximum-likelihood (ML) phylogeny of 18 species of the family Ixodidae based on the 13 concatenated nucleotide sequences of protein-coding genes (PCGs), utilizing Tamura-Nei model and after 1,000 bootstrap replications. Limulus polyphemus (NC003057) was used as the outgroup. The black square sign represents the species in this study. Bootstrap support values are shown above the nodes.

## Data Availability

The data that support the findings of this study are openly available in GenBank of NCBI at https://www.ncbi.nlm.nih.gov/, reference number MW411447.
